# A single reporter mouse line for Vika, Flp, Dre, and Cre-recombination

**DOI:** 10.1038/s41598-018-32802-7

**Published:** 2018-09-27

**Authors:** Madina Karimova, Oliver Baker, Aylin Camgoz, Ronald Naumann, Frank Buchholz, Konstantinos Anastassiadis

**Affiliations:** 1 Medical Systems Biology, UCC, Medical Faculty Carl Gustav Carus, Technische Universität Dresden, 01307 Dresden, Germany; 2 Stem Cell Engineering, Biotechnology Center, Technische Universität Dresden, 01307 Dresden, Germany; 3 Max Planck Institute of Molecular Cell Biology and Genetics, 01307 Dresden, Germany; 4 Present Address: Georg Speyer Haus, Institute for Tumor Biology and Experimental Therapy, 60596 Frankfurt am Main, Germany; 5 Present Address: King’s College London, Guy’s Campus, SE1 1UL, London, United Kingdom

## Abstract

Site-specific recombinases (SSR) are utilized as important genome engineering tools to precisely modify the genome of mice and other model organisms. Reporter mice that mark cells that at any given time had expressed the enzyme are frequently used for lineage tracing and to characterize newly generated mice expressing a recombinase from a chosen promoter. With increasing sophistication of genome alteration strategies, the demand for novel SSR systems that efficiently and specifically recombine their targets is rising and several SSR-systems are now used in combination to address complex biological questions *in vivo*. Generation of reporter mice for each one of these recombinases is cumbersome and increases the number of mouse lines that need to be maintained in animal facilities. Here we present a multi-reporter mouse line for loci-of-recombination (X) (MuX) that streamlines the characterization of mice expressing prominent recombinases. MuX mice constitutively express nuclear green fluorescent protein after recombination by either Cre, Flp, Dre or Vika recombinase, rationalizing the number of animal lines that need to be maintained. We also pioneer the use of the Vika/vox system in mice, illustrating its high efficacy and specificity, thereby facilitating future designs of sophisticated recombinase-based *in vivo* genome engineering strategies.

## Introduction

Site-specific recombinases are instrumental genetic tools used in a variety of applications, including synthetic biology^1^, conditional mutagenesis^2^ and lineage tracing^3^. SSRs catalyze recombination between two recognition target sites and can be grouped into 2 families - serine and tyrosine recombinases, according to their amino acid residue that attacks specific phoshodiesters to cleave the DNA strands^4^. In particular, the tyrosine recombinase Cre/loxP system is widely utilized in mammalian model organisms, due to its ease of use and efficacy/specificity to recombine its 34 bp loxP-target *in vivo*. Numerous Cre mouse-lines have been generated that express the enzyme from a large variety of tissue-specific or inducible promoters^5,6^. The “Cre-Zoo” has transformed mouse genetics and conditional gene-knockouts and lineage tracing are now possible in virtually any organ or tissue^2^. Furthermore, the Cre-lines are instrumental to model human diseases in mice^7,8^.

Many genetic alteration strategies, for instance the consecutive deletion of several genes in different tissues or sequential cell labeling, require more than one recombinase system to efficiently function *in vivo*^9,10^. The tyrosine recombinase Flp from the yeast Saccharomyces cerevisiae 2 μ plasmid recombines 34 bp FRT target sites and was discovered around the same time as the Cre/loxP system^11^. Indeed, the Flp/FRT system has also shown activity in mammalian cells^12^, albeit at much reduced efficiency due to instability of the enzyme at temperatures relevant for mammalian cells^13^. The isolation of more thermostable Flp mutants^14^ and mammalian codon optimization^15^ has greatly improved the utility of Flp recombinase for research in mammalian model organisms^16,17^ and the Flp/FRT system is now frequently used orthogonally together with the Cre/loxP system in mice^18–20^.

More recently, the Dre/rox system was discovered in the bacteriophage D6^21^ and shown to function efficiently in mice^22^. Dre has considerable homology to Cre and its target site, rox, also only differs to loxP in three out of thirteen nucleotides per half-site^21^. Despite this high homology, cross-recombination between the two systems is typically not observed^9,22,23^, although some groups have reported mild cross-reactivity upon high expression of the recombinases^24,25^. Consequently, successful concurrent use of Cre and Dre^9,10,26^ and Cre, Flp and Dre^23^ in mice has been demonstrated.

While the above three SSR systems allow for sophisticated genome engineering in mice, such as sequential genetic intersectional labeling^23^, many advanced approaches would require additional orthogonal SSRs. Actually, several additional Flp-like^27,28^ and Cre-like^29–31^, recombination systems have been discovered, but none of these systems has been tested for their efficacy and specificity in transgenic mice, yet.

We set out to expand the scope of SSR systems for orthogonal use in mice and to streamline the analysis of recombinase expressing mice by generating a mouse line carrying a quadruple-recombinase-responsive reporter allele. The multi-reporter mouse line for loci-of-recombination (X) (MuX) will be useful to characterize recombination characteristics of mice expressing either Cre, Flp, Dre or Vika recombinase and reduce the number of mice required to be maintained in animal facilities.

## Results

### Choice of additional SSR system for use in mice

A panel of new yeast and bacteria-derived tyrosine recombinases and their respective target sites have been discovered and characterized *in vitro* (reviewed in^32^). We first considered published characteristics of recombinases to nominate the most promising additional SSR system to be used in conjunction with the established systems for high efficiency and specificity recombination in mice. The recombinases KD, B2, B3 and R^28^, like Flp, are all derived from yeast. Yeasts typically grow at temperature at or below 30 °C. This fact explains why Flp shows low activity at temperatures relevant to mice^13^ and why it was necessary to genetically engineer this enzyme for efficient use in mammalian organisms^14,16^. KD and B3 recombinases have been successfully tested via transient plasmid in utero electroporation in mouse embryos^28^. However, the possibility that these and other yeast-derived recombinases would also have reduced activity at 37 °C was considered to be high. Hence, they were not further pursued for the generation of transgenic mice. The recombinases SCre and VCre have shown activity in Medaka^33^ and they have also demonstrated their utility in mammalian cells^29^. However, recombination assays in bacteria have shown that they are less active in comparison to other SSR systems^25,30,31^. Likewise, Nigri recombinase was shown to recombine its substrate, nox, at lower efficiency in bacteria^31^. Panto recombinase showed similar recombination kinetics to Dre and Cre in bacteria, but, like VCre^25^, Panto was shown to posses cross-recombination activity on target sites of another recombinase^31^. In contrast, the Vika/vox system has demonstrated to function with high activity in bacteria^25,30^, yeast^34^ and in mammalian tissue culture cells^30,31^ and importantly, no cross-recombination with other SSR-systems, or cytotoxicity upon overexpression in mammalian cells has been observed^30^. We therefore decided to choose the Vika/vox system for use in mice.

### Vika/vox functions in mES cells

**Figure 1:**
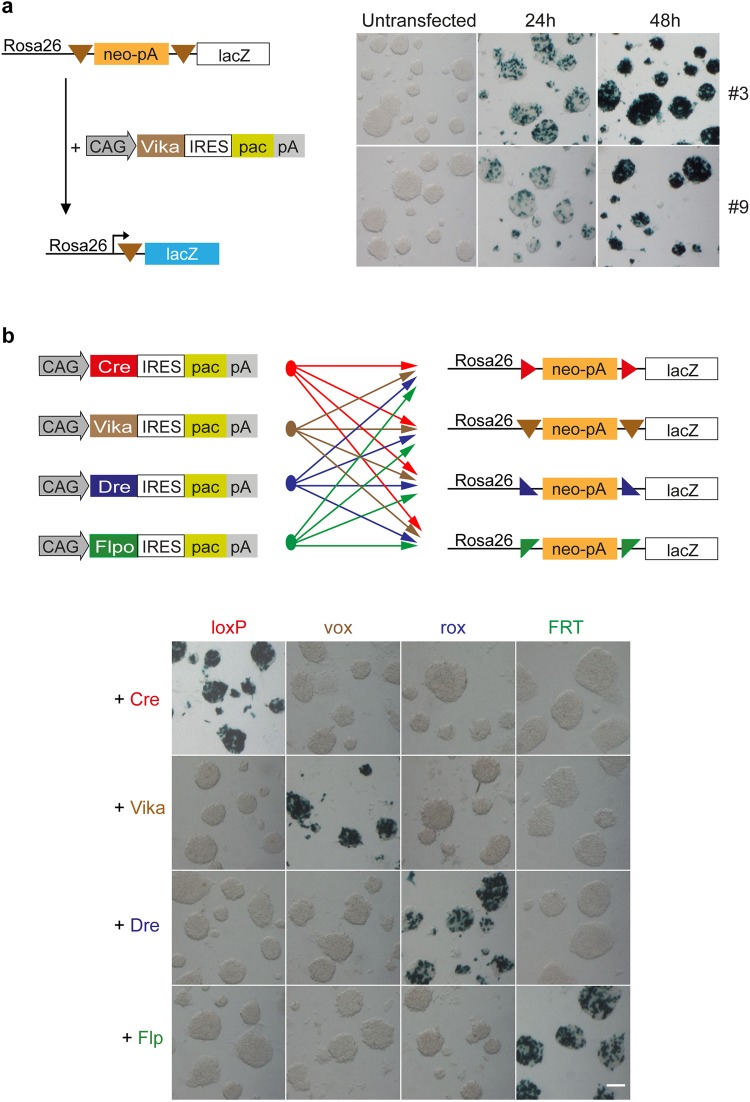
Vika recombines specifically its target sites (vox) in mES cells. (**a**) Schematic diagram of the Rosa26-vox-neo-vox-lacZ reporter before and after Vika recombination. Two mESC clones (#3 and #9) with stably integrated lacZ reporters were transiently transfected with CAG-Vika-IRES-puro and analysed at 24 h and 48 h by lacZ staining. (**b**) Each of the four recombinase expression vectors (3 µg each per 6-well) were transfected in the four stable Rosa26 reporter mES cells lines. Cells were analysed 48 h after transfection by lacZ staining. (Scale bar = 100 µm).

Before generating a Vika mouse, we further investigated the applied properties of this recombinase. In order to compare Vika to other established site-specific recombinase systems in mouse embryonic stem (mES) cells, we generated a similar reporter vector equivalent to the reporter vectors that have been used for Cre, Flp and Dre recombinases^17^. A neomycin-stop (neo-pA) cassette was flanked with vox sites and cloned upstream of the lacZ gene into the Rosa26 targeting vector generating Rosa26-vox-neo-pA-vox-lacZ (Rosa26-vox-lacZ). This reporter was targeted to the ubiquitously expressed Rosa26 locus in mES cells^35^. Two correctly targeted clones (#3 and #9) identified by Southern blot analysis (Supplementary Fig. S1a shows clone #3) were subsequently transfected with a mammalian codon-optimized Vika expression plasmid. Vika activity leads to the excision of the neo-stop cassette and expression of the lacZ gene from the Rosa26 promoter. Both clones showed prominent and similar recombination efficiencies after 24 h and 48 h, demonstrating that the Vika/vox system functions well to recombine vox sites placed in the genome of mES cells (Fig. 1a).

### Vika efficiently recombines vox-sites in mES cells and displays no cross-reactivity with other SSR systems

For Vika to become a valuable tool for future *in vitro* and *in vivo* applications, cross-reactivity with other SSR systems have to be excluded, while recombination on vox sites has to be achieved at high efficacy. Therefore, Vika was tested on previously established Rosa26-loxP-, Rosa26-rox-, and Rosa26-FRT-lacZ mESC reporter lines^17^. Vice versa, Cre, Dre, and Flpo were tested on the Rosa26-vox-lacZ reporter cells to evaluate any potential recombination activity. Vika did not recognize loxP, rox, and FRT sites indicated by the absence of β-galactosidase staining when transiently transfected into the respective lacZ reporter lines (Fig. 1b). Moreover, no recombination activity on vox sites was detectable with Cre, Dre, and Flpo recombinases. Remarkably, Vika displayed recombination efficiencies comparable to Cre, Dre and Flpo resulting in almost complete recombination after 48 h (Fig. 1b).

### Generation of Vika deleter mice

The results obtained in mES cells confirmed the adequate characteristics of Vika/vox for use in mice. We therefore proceeded with the generation of Vika deleter mice (for overview see Supplementary Fig. S1b). For this purpose we electroporated R1 mESCs with a linearized CAG-Vika-IRES-puro expression vector, where Vika is expressed from the ubiquitous CMV early enhancer/chicken beta actin (CAG) promoter^36^. Puromycin resistant colonies were expanded and analysed by Southern blot for single copy integration (data not shown). Several clones were transiently transfected with a Vika reporter plasmid to confirm expression of the recombinase (data not shown) and two positive clones were injected into blastocysts. Chimeras were back-crossed to C57Bl6/JHsdOla wt females to establish a line (CAG-Vika) that transmits the Vika coding sequence through the germline. The mice could be bred to homozygosity assessed by mating the progeny of a CAG-Vika intercross and observing the frequency of transgene transmission.

### Generation and testing of the MuX construct

**Figure 2:**
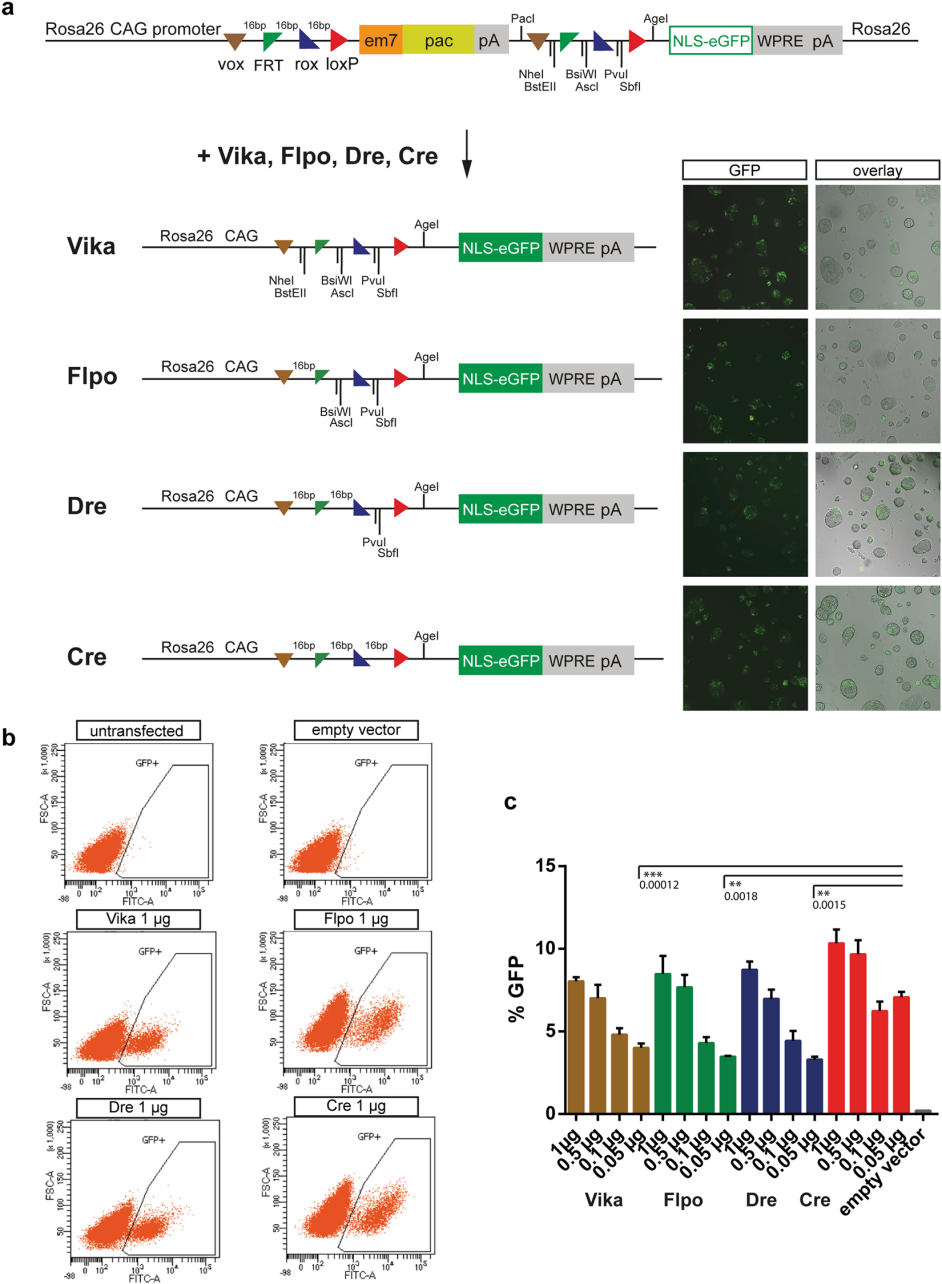
The multi site reporter vector is functional in mESCs. (**a**) Schematic diagram of the Rosa26-multi site reporter (MuX) before and after Vika, Flpo, Dre and Cre recombination. Stable mESC reporter clones were transiently transfected with expression vectors and analysed at 48 h for GFP expression. (**b**) Flow cytometry to measure GFP levels after transfecting different amounts of the recombinase expression vectors (0.05 to 1 µg per 6-well). Representative plots of cells transfected with 1 µg recombinase expression vector are shown. (**c**) Quantification of (**b**). Error bars represent standard deviations. Statistical significance is shown only for P < 0.01 (**) and P < 0.001 (***).

After generation of the Vika deleter mice, we needed to validate its performance *in vivo*. Consequently, we had to establish a new vox-reporter line. Instead of making a single vox-reporter mouse, we decided to generate a multi-reporter mouse line for loci-of-recombination (X) (MuX) for four tyrosine recombinases (Cre, Dre, Flp and Vika) that would allow rapid and streamlined characterization of the different recombinases in the same reporter strain (Fig. 2).

To this end, the four recombinase targets (RTs; vox, FRT, rox and loxP) were each placed in a head-to-tail orientation flanking the puromycin acetyltransferase - polyA (pac-pA) cassette followed by the coding sequence for enhanced green fluorescent protein (eGFP), tagged with a nuclear localization signal (NLS). The RTs that were upstream to the puromycin stop cassette were separated by 16 random bases (16 bps), whereas single restriction enzyme target sites were placed between the downstream RTs to facilitate genotyping after recombination (Fig. 2a). To confirm that expression of eGFP is not compromised by the array of the four RTs positioned between the CAG-promoter, we tested the versions of Cre, Flp, Dre or Vika recombined constructs, respectively. Recombination *in vitro* was accomplished by transforming the reporter vector together with the respective recombinase into *E.coli*. Pre-recombined vectors were then transiently transfected into HeLa cells and assessed for eGFP expression. All recombined versions of the constructs led to comparable eGFP expression, demonstrating the functionality of the approach (Supplementary Fig. S2.1). The recombined products were PCR amplified across the RTs and sequenced. Each recombinase generates a specific genetic fingerprint, which was confirmed by sequencing (Supplementary Fig. S2.2). As a next step, we co-transfected HeLa cells with the MuX reporter vector and with an expression plasmid for each of the four recombinases, respectively. The transfected cells expressed eGFP, demonstrating robust activity of all tested recombinases (Supplementary Fig. S2.3). Hence, the MuX construct functions to report recombination induced by any of the four recombinases.

Based on the positive co-transfection results, we targeted the MuX construct to the Rosa26 locus by electroporation in R1 mES cells. For functional testing, one correctly targeted clone was transiently transfected with different amounts of expression plasmids (0.05 to 1 µg per 6-well) for each of the four recombinases. Fluorescence microscopy (Fig. 2a) and flow cytometry analyses revealed comparable numbers of eGFP-positive cells for all tested conditions, validating the functionality of the MuX reporter and demonstrating high activity for all tested recombinases (Fig. 2b,c).

### Generation and analysis of MuX mice

Mice were generated by injection of the MuX R1 mES cells into 8-cell stage embryos, followed by transfer into pseudopregnant donors. Germ line transmission was obtained by mating male chimaeras with C57Bl6/JHsdOla wt females. To benchmark MuX mice to other fluorescent reporter mice, we crossed Rosa26-eYFP^37^, Z/EG^38^ and MuX reporter mice to the PGK-Cre deleter strain^39^. Analysis of E6.5 embryos by fluorescence microscopy revealed prominent expression of the fluorescent proteins in virtually all cells in the eYFP and MuX reporter mice, whereas GFP expression from the Z/EG reporter was detected in all cells of the embryo, but expression was lower in cells of extra-embryonic tissues (Supplementary Fig. S3.1). In contrast to the eYFP reporter, individual cells that had undergone recombination in the MuX mice were easily distinguishable, due to the nuclear expression of GFP in these mice (Supplementary Fig. S3.1). At E10.5 expression of the fluorescent protein was homogenously expressed in embryos from all three reporter lines (Supplementary Fig. S3.2). As in tissue culture cells, the array of the 4 recombination target sites in front of GFP did not interfere with expression levels in the embryos. Hence, MuX mice provide a useful Cre-reporter line that marks all cells that have undergone recombination through nuclear expression of GFP.

**Figure 3:**
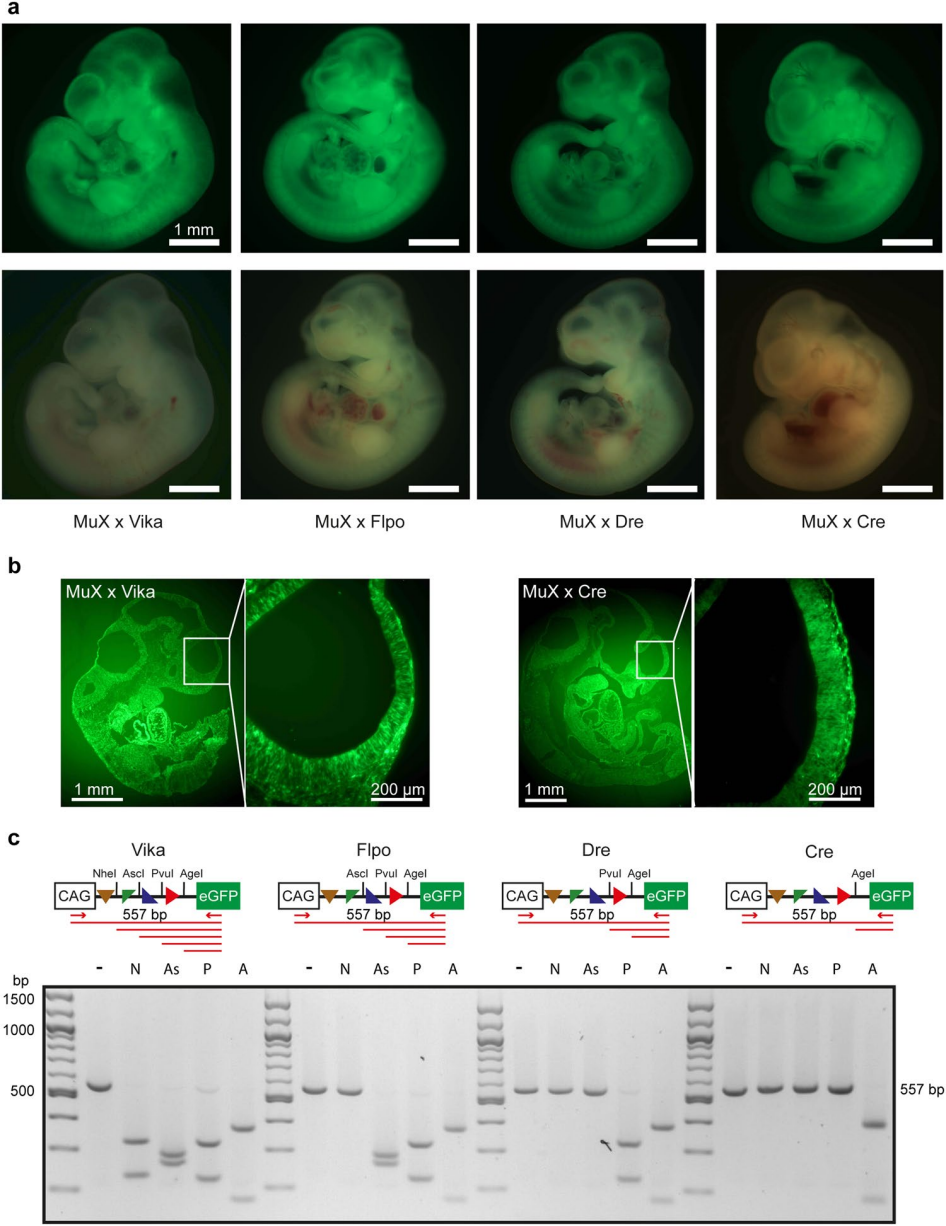
The MuX reporter is functional in mice. (**a**) Micrographs showing embryos at E10.5 isolated from intercrosses between Vika, Flpo, Dre and Cre deleter male mice with Rosa26-VFRL-eGFP (MuX) reporter mice. The reporter protein is ubiquitously expressed. Scale bar = 1 mm. (**b**) Sections of E10.5 embryos. (**c**) PCR product from each of the four deleter x MuX reporter crosses followed by digestion with indicated restriction endonucleases (− = undigested, N = NheI, As = AscI, P = PvuI, A = AgeI).

We next crossed MuX mice to Cre-, Dre-, Flpo- and Vika-deleter mice and analysed GFP expression in E10.5 embryos. All embryos showed ubiquitous expression of GFP at comparable fluorescence intensities, demonstrating that MuX mice are equally suitable for all four recombinases (Fig. 3a). To test whether recombination was complete and has occurred in all cells of the embryo, we embedded the embryos in OCT and performed sagittal cryosections. GFP was homogenously expressed in all parts of the embryo (Fig. 3b). Recombination was assayed by PCR using primers located upstream and downstream of the recombination target site array (Fig. 3c). PCR products were digested with restriction enzymes that are specific for each recombination outcome. This simple genetic fingerprinting assay enabled us to identify on the molecular level the site-specific recombinase that was active (Fig. 3c).

To demonstrate lack of cross-reaction of Vika on loxP, rox and FRT sites *in vivo*, we crossed Vika deleter mice to Cre-reporter (Z/EG^38^), FLP-reporter (RC::Fela^40^) and Dre-reporter (Rosa-rox-lacZ^22^) mice. As positive controls we crossed each deleter line to its corresponding specific reporter. We isolated embryos at E10.5 and checked for the expression of the reporter protein. While efficient recombination was apparent in the positive control crosses, no GFP (Z/EG, RC::Fela) or lacZ (Rosa-rox-lacZ) positive cells were observed when Vika mice were crossed to any of the orthogonal reporters (Supplementary Fig. S3.3). Likewise, PCR assays demonstrated lack of recombination when Vika were crossed to Z/EG, RC::Fela or Rosa-rox-lacZ mice (Supplementary Fig. S3.3). Hence, Vika is highly specific for recombining vox-sites and does not act on the target sites of other recombinases *in vivo*.

**Figure 4:**
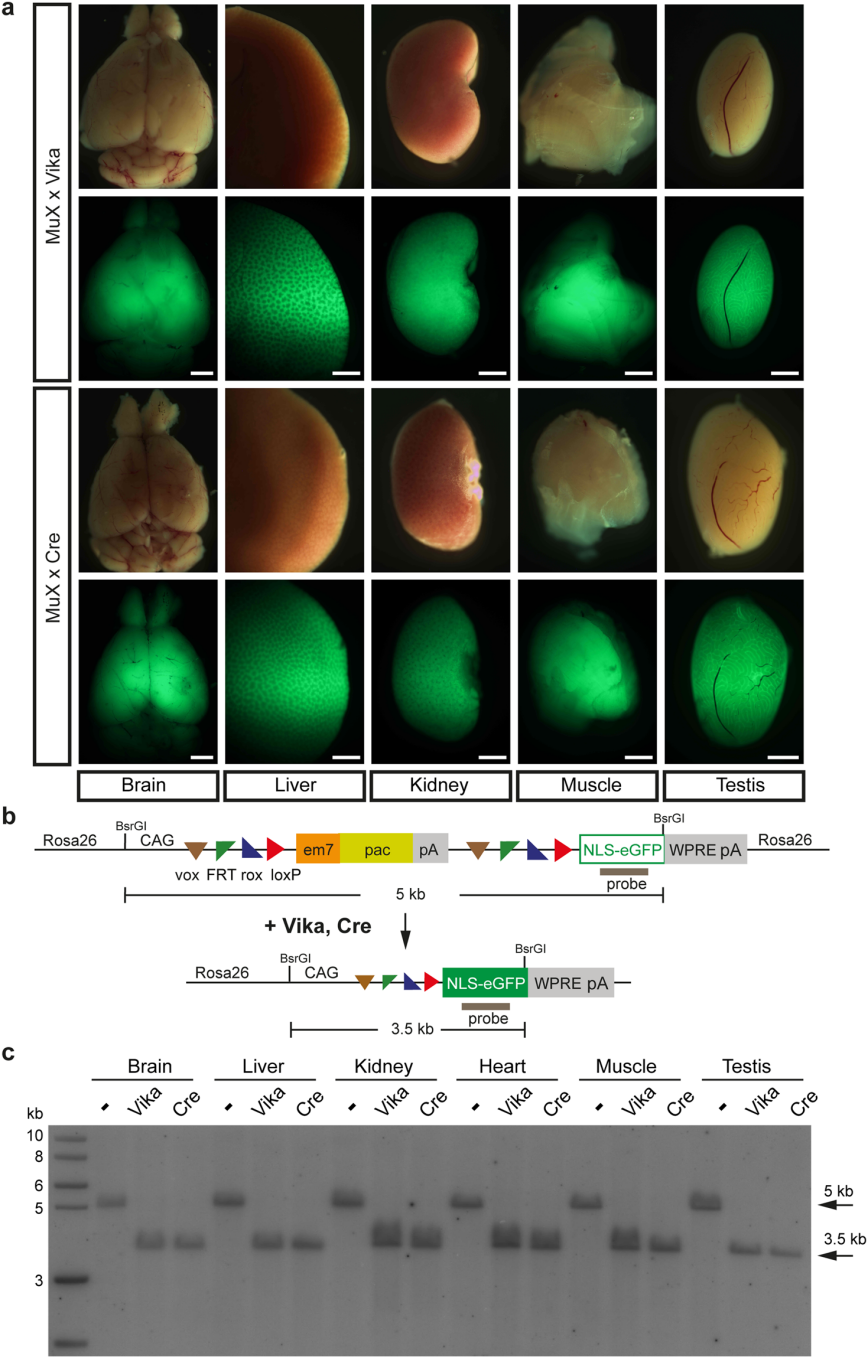
Vika-recombination is complete in adult tissues. (**a**) Organs isolated from MuX × Vika and MuX × Cre mice and analysed for GFP expression. Scale bar = 1 mm. (**b**) Schematic diagram of the Southern strategy for detecting recombination. (**c**) Southern blot showing complete recombination in all analysed tissues.

To assess if GFP expression of the MuX reporter is maintained in adult mice we examined different organs from crosses of Cre- or Vika deleters with MuX mice. Expression of GFP in all analysed organs (brain, liver, kidney, muscle, testis) was homogenous and levels were comparable between Cre and Vika recombinases (Fig. 4), demonstrating the utility of the MuX reporter to analyse recombination in adult mice. Complete recombination was detected on the molecular level using Southern blot (Fig. 4b,c). To detect complete recombination at the single cell level we imaged cryosections of the liver. GFP was homogeneously expressed and localized in the nucleus of liver cells, whereas there was no GFP expression in the absence of recombinase (Supplementary Fig. S4.1a). Moreover, recombination was virtually complete in the peripheral blood and bone marrow of Cre x MuX mice as detected by flow cytometry (Supplementary Fig. S4.1b).

To test recombination efficiency in adult MuX reporter mice, we crossed the mice to the tamoxifen inducible Rosa26-Cre-ERT2 strain^41^. In parallel we also crossed the Rosa26-Cre-ERT2 strain to the Rosa26-eYFP reporter (Supplementary Fig. S4.2a shows a diagram of the crossings). Double knock-in (KI) mice (Rosa26-Cre-ERT2 (RC)::MuX = [RC:MuX] or Rosa26-Cre-ERT2::Rosa26-eYFP = [RC/RY]) were treated at the age of 22 weeks with tamoxifen as described^42^. Recombination efficiency was analysed by Southern hybridization analysis using a GFP probe and DNA from testis and liver from single reporters (Rosa26-eYFP = RY/+) and double KI mice (Supplementary Fig. S4.2b). As expected, no recombination was detected in the absence of tamoxifen. In contrast, recombination was observed to a similar extent between the two reporter strains when the mice were treated with tamoxifen. However, recombination was not complete, probably because the treatment protocol was suboptimal for this age of mice.

We conclude that the Vika/vox system is suitable for orthogonal use with at least three other recombinase systems in mice and that the MuX mice are well suited as recombinase-reporter mice for Cre, Dre, Flp and Vika recombinases.

## Discussion

SSRs have become an integral part in the genetic toolbox of biologists and biomedical scientists. For the mouse, the Cre/loxP system is the most widely used SSR for advanced genome engineering^32^. But with increased sophistication of experimental designs, the Dre/rox system and enhanced versions of Flp recombinase are now also routinely used in mice, with a number of Dre and Flp-deleter strains^16,17,22,43,44^ and tissue-specific Dre-drivers^10,26,45^ as well as tissue-specific Flp-drivers^46–51^ demonstrating the utility of these enzymes *in vivo*. As an example, combinatorial use of tissue specific Dre and Cre recombinases in mice revealed that liver vasculature arises from the endocardium^26^. Even more complex experimental designs are emerging, calling for additional SSR systems that operate efficiently and specifically in mammals. We demonstrate that the Vika/vox system is an additional SSR system suitable for robust recombination in mice. The Vika-deleter demonstrates full recombination without cross-recombination on loxP, FRT or rox target sites, making Vika/vox a versatile orthogonal recombinase system for use in mice. The Vika-deleter should be useful to excise genes that are flanked by vox sites in all cells of the organism. Given the comparable properties of Vika to other recombinases *in vivo*, we expect that expression of Vika from tissue-specific promoters should result in efficient conditional mutagenesis in animals. It will therefore be worthy to generate tissue-specific Vika-driver strains and evaluate the performance of Vika in this setting. Because SSR systems that function well in mice have proven useful in other model organisms^52,53^, we predict that the Vika/vox system will also work well in other species.

Conditional mutagenesis and lineage tracing (cell fate mapping) were progressively made more predictable and reliable with the emergence of new site-specific recombinases. The conditional mutagenesis strategy based on the multi-purpose allele design^54^, requires Flp recombinase for excising the selection reporter cassette and Cre for deleting the frame shifting-exon. More recent targeting vector designs have incorporated an additional selection cassette containing a different antibiotic resistance gene driven by a constitutively expressed promoter and flanked by rox-site for Dre recombination^55^. Having now more than three site-specific recombinases that function efficiently and specifically in mice, one can envision more complicated applications either for conditional mutagenesis or for lineage tracing (cell fate mapping). For example, separate genomic regions encoding different protein domains could be flanked by independent recombination target sites and excised sequentially. One or more exons coding for a specific protein domain can be flanked by Cre/loxP, whereas others by Vika/vox and thus allow a precise analysis on the function of each protein domain by deleting them in the same genetic background, whilst the other two recombinases (e.g. Flp/FRT and Dre/rox) can be reserved for excising selection cassettes. Similar logic can be applied to dissect functions of several long non-coding RNAs or genes with high homologies by excising the critical sequences with orthogonal SSR systems. In cell fate mapping studies, one could take advantage of cell-type specific gene expression signatures and use them to drive recombinase expression indicated by a reporter system. However, expression of the driver gene can be broad and there is requirement of an additional gene specific driver in order to label a subpopulation of cells. Those so-called genetic intersectional labeling strategies have been mainly conducted by combinatorial use of two SSRs, either Flp and Cre or Dre and Cre^9,10,18,19^. The sequential use of three recombinases (Cre, Flp and Dre) enabled for the first time the labeling of subpopulations of noradrenergic neurons^23^. Consequently, the use of 4 recombinases and sophisticated reporters will contribute to the discovery of new cell populations in different tissues. Furthermore, complicated genomic manipulations, including Recombination Mediated Cassette Exchange^56^, conditional mutagenesis based on inversions of mutagenic cassettes (Conditionals by Inversion (COIN) and Flip-excision (FlEx) methods)^57,58^ or chromosomal translocations^59^ can be better controlled by the combinatorial use of a panel of different SSRs.

An important criterion for the combined use of different recombinases in one organism is target-site specificity and cross-reaction assays are important for choosing from a list of SSRs the ones that can be efficiently combined *in vivo*. The recombinase Panto derived from Pantoea sp. aB recombines not only its native target-site called pox, but also the rox site for Dre recombination^31^. Furthermore, partial cross-reaction has been reported between R and B2 recombinases in Drosophila^28^. Therefore, these recombinase systems should not be used in combination when specificity is a requirement. In contrast, Cre- and Flp-recombinases together with alleles carrying their specific target sites have been used in the same system without any cross-recombination^18,60^. We and others have shown that no cross-reaction was observed with respect to site specificity between Cre and Dre recombinases in *E. coli* as well as in mammalian cells and in mice^9,10,22,45^. Similar results were obtained in Zebrafish in which a Dre-reporter was not recombined by Cre and vice versa^61^. In contrast, Fenno *et al*. have reported cross-reaction between Dre and Cre when the recombinases were expressed from viral vectors *in vitro* and *in vivo*^24^. It is therefore possible that the two systems cross-react when the recombinases are expressed at very high levels. In our experimental setting we did not observe any cross-reaction between Cre, Flp, Dre and Vika recombinases and their target sites, indicating that these recombinase systems can successfully be employed in combination.

Increasing the number of different recombinases for use in mice requires the generation of reporter animals to characterize the recombinase-driver strains. Indeed, a panel of different recombinase-reporter strains for Cre, Flp and Dre have been reported^22,37,38,40,62^. The number of Cre-driver mice is constantly increasing^5,6^ and a number of tissue-specific Dre-drivers^10,45^ as well as tissue-specific Flp-drivers^46–51^ also already exist. The prediction is that more will be made and that Vika will likely follow this trend. Therefore, the need to have reliable reporter mice for the easy and rapid characterization of newly generated recombinase-driver lines is of utmost importance. In this respect, it will be more reasonable and cost-effective to breed only one mouse line that can serve as a reporter for 4 different recombinases, rather than keep a specific reporter line for each recombinase. To circumvent the necessity to maintain separate recombinase-specific reporter lines in the animal facility, we have generated the multi-site reporter strain MuX. The MuX line is intended for testing recombination efficiency of tissue specific or inducible Cre, Flp, Dre or Vika recombinase mouse lines and expresses nuclear GFP in all cells of the organism after recombination. Reporter mice that testify sequential Cre and Flp recombination have been generated^40,46,62,63^, but these lines are more suitable for lineage tracing studies. Through the incorporation of a unique restriction enzyme panel between the recombinase target-sites, MuX mice are in principle also useful to investigate which target-site was recombined first. Therefore, MuX mice might be useful for lineage tracing. However, another set-up employing an array of different fluorescent markers each flanked by either loxP, rox, FRT or vox target-sites is likely better suited for such endeavors. With the possibility to combine four different recombinases in one animal, many innovative developmental and biomedical applications are on the horizon.

## Methods

### DNA constructs

Vectors were build using standard or recombineering^64^ methods. Oligonucleotides are shown in Supplementary Table S1 and cloning details are available upon request. In brief, codon-optimized Vika sequence including nuclear localization signal (NLS) was PCR amplified with primers containing EcoRI restriction sites (Vika-Fwd and Vika-Rev) from pNPK-NLS-Vika^30^ and cloned into pCAG-(EcoRI)-IRES-puro, a derivative of pCAG-Flpe-IRES-puro expression plasmid^65^. Rosa26-vox-neo-pA-vox-lacZ reporter was generated by amplification of em7-neo-pA from pR6K-amp-PGK-em7-neo-pA template using the oligonucleotides Rosa-vox-neo-fwd and Rosa-vox-neo-rev and inserted by recombineering into Rosa26-loxP-lacZ targeting vector (a derivative of Rosa26-loxP-neo-loxP-lacZ^17^ after Cre-recombination in *E.coli*). The multi-site reporter construct was assembled by gene synthesis of a puromycin cassette and standard cloning methods, using pCAG-eGFP expression vector as a starting point. Briefly, a cassette consisting of a mammalian codon optimised puromycin resistance gene with synthetic polyadenylation signal positioned between two arrays of recombination sites (one array = vox, FRT, rox, loxP = VFRL) was assembled via gene synthesis (Geneart, Thermo Fisher Scientific). The cassette contained six unique restriction sites in the array downstream of the puromycin resistance gene to permit RFLP analysis of post-recombination events. Restriction sites were placed in the following manner: vox - NheI - BstEII - FRT - BsiWI - AscI - rox - PvuI - SbfI - loxP. The region between the recombination target sites in the upstream array corresponding to restriction sites has been replaced by random 16 bp sequences. Puromycin cassette was cloned via AgeI and EcoRI into backbone vector, between CAG promoter and eGFP-SV40pA. A nuclear localisation signal (nls) was added to an amino-terminal end of eGFP via PCR utilising primers nls-eGFP-up and eGFP-BsrGI-low via AgeI and BsrGI cloning. The Woodchuck Hepatitis Virus Posttranscriptional Regulatory Element (WRPE) signal was PCR amplified using primers WPRE-up and WPRE-low from Ai9 plasmid^66^ and cloned via BsrGI + BsiWI into the reporter. As a next step the “CAG-VFRL-puro-VFRL-nls-eGFP-WPRE” cassette was cloned via recombineering into a modified Rosa26 targeting vector (Rosa26-5′HA-CAG-ccdB-amp-Rosa26-3′HA). The final construct “Rosa26-CAG-VFRL-puro-VFRL-nls-eGFP-WPRE” was designated as Rosa26-multi site reporter (MuX).

### Reporter validation and Cell culture

Reporter plasmids recombined by either of four recombinases Vika, Flp, Dre or Cre were generated in order to validate efficient GFP expression and absence of an obstructing effect of an array upstream of the GFP ORF. Briefly, reporter plasmid (before addition of nls and WPRE) and plasmid for bacterial expression of recombinases R6K-Vika, R6K-Flp, R6K-Dre or R6K-Cre^30^ were co-transformed in *E.coli* DH10B. *E.coli* clones were cultured overnight in 5 ml liquid culture under double selection kanamycin (reporter) and tetracycline (R6K) to allow co-culture of both plasmids. DNA was isolated according to manufacturers protocol (QiaPrep Spin miniprep kit, Qiagen) and retransformed into *E.coli*. One random bacterial colony from each plate was picked and plasmid DNA was isolated. Constructs after recombination are referred to as *E.coli* pre-recombined. Pre-recombined reporters were used for transfection into HeLa Kyoto cells cultured at 37 °C and 5% CO_2_ and analysed 48 h post-transfection for fluorescence; pCAG-eGFP plasmid served as a positive control. HeLa cells were plated at a density of 4 × 10^4^ cells per well in 24-well dishes and cultured in 4.5 mg/ml glucose Dulbecco’s Modified Eagle’s Medium (DMEM, Gibco-Invitrogen), supplemented with 10% fetal bovine serum (Invitrogen), 100 U/ml penicillin and 100 mg/ml streptomycin (Gibco-Invitrogen). At 80–90% confluency, cells were transfected with the pre-recombined reporter plasmids using Lipofectamine 2000 Transfection Reagent (Invitrogen) according to manufacture’s instructions. Growth media was changed 4 h post transfection and cells were imaged for GFP expression 48 h after transfection with an EVOS microscope (Thermo Fisher Scientific). MuX reporter containing NLS and WPRE elements was tested in HeLa cells as above. For that MuX reporter, was co-transfected with recombinase expression plasmids NPK-NLS-Vika, NPK-NLS-Flpo, NPK-NLS-Dre or NPK-NLS-Cre^30^. Cells were observed at 48 h post-transfection for nuclear GFP.

Detailed protocols for culture and manipulation of mouse embryonic stem cells (mESCs) have been previously described^67^. In brief R1 mESCs were cultured on mitomycin C-inactivated mouse embryonic fibroblasts using 4.5 mg/ml glucose DMEM supplemented with 15% fetal calf serum (FCS), 2 mM L-glutamine, 1 mM sodium pyruvate, 1% penicillin/streptomycin, 100 μM non-essential amino acids (all from Invitrogen), 100 μM β-mercaptoethanol (Sigma) and leukemia inhibitory factor. mESCs (5 × 10^6^) were electroporated with 40 μg linearized targeting vector (Rosa26-vox-neo-pA-vox-lacZ and Rosa26-CAG-vox-FRT-rox-loxP-puro-pA-vox-FRT-rox-loxP-GFP) using a Bio-Rad electroporator (250 V, 500 μF, 4 mm cuvette) and selected for 7 days with 200 μg/ml G418 (Invitrogen) or 1 μg/ml puromycin (Sigma), respectively. Resistant mESC colonies were picked into 96-well plate, expanded, and analyzed by Southern blot. For transient transfections, R1 mES cells were seeded one day prior to transfection in 6-well plates at a density of 2 × 10^5^ cells/well. The following day, media was changed and transfected using Lipofectamine LTX according to manufacture’s instructions (Invitrogen). Unless otherwise indicated the cells were transfected with 3 µg DNA per 6-well. Media was changed the following day and cells were incubated for another 24 h and then analysed either by Flow cytometry or lacZ staining. Statistical significance was calculated using the paired two-tailed Student’s *t* test.

### Southern blot

Genomic DNA was extracted from cells or tissues using lysis buffer (10 mM Tris pH:8.0, 150 mM NaCl, 10 mM EDTA, 0.1% SDS) containing 0.1 mg/ml proteinase K, followed by phenol/chloroform purification and ethanol precipitation. DNA was cut overnight with the indicated enzyme, separated on 0.8% agarose gels and blotted to nylon membranes. Probes were labeled with ^32^P by random priming (Roche Diagnostics) and hybridized using the Church and Gilbert protocol.

### Generation of Vika deleter and MuX reporter mice and their validation

pCAG-Vika-IRES-puro was linearized with SpeI and electroporated into R1 ES cells as described above. Two clones with single copy integrations were delivered by laser-assisted injection into 8-cell stage embryos^68^. Similarly, one mESC clone correctly targeted with the multi-site reporter MuX in the Rosa26 locus was injected into 8-cell embryos. Chimeras were crossed to C57Bl6/JHsdOla mice and screened by Southern blot for germline transmission. The established lines were genotyped by PCR using primers listed in Supplementary Table S2. The new generated lines were intercrossed to validate their functionality and further crossed with other existing deleter or reporter lines to analyse their specificity. The isolated embryos (E10.5) were imaged with an Olympus MVX10 macro fluorescence microscope. Embryos were genotyped by PCR (primers are in Supplementary Table S2). All experiments involving the generation of mice were performed according to German law by the Transgenic Core facility at the MPI-CBG, Dresden. For inducing recombination in adult mice the Rosa26-Cre-ERT2 line^41^ was crossed to the Rosa26-eYFP and to the MuX reporter. Tamoxifen (Sigma, T5648) was administered in adult mice by oral gavage as described^42^. In brief, male mice received a daily dose of 4.5 mg for 4 consecutive days (3 days for females) followed by 3 days break and another 2 days gavage. Tamoxifen experiments were performed according to German law and approved by the relevant authorities Landesdirektion Sachsen (Permit number: TVV 41/2016).

### β-galactosidase staining

Cells were rinsed with PBS and fixed with 2% Formaldehyde and 0.1% Glutaraldehyde for 2 minutes, washed three times with PBS, incubated O/N at 37 °C with PBS containing 2 mM MgCl_2_, 1.6 mg/ml Potassium Ferricyanide, 2.1 mg/ml Potassium Ferrocyanide, and 1 mg/ml X-Gal. Cells were washed once with PBS and imaged with a stereomicroscope (Nikon SMZ 1500).

### Flow Cytometry

Cells were trypsinised, washed with PBS and fixed with 2% formaldehyde in PBS for 20 min on ice. Cells were centrifuged (1000 rpm, 5 min, RT), washed once with PBS, centrifuged again and the pellet was resuspended in 1 ml PBS. Measurements were performed with a BD FACSCanto™ II (BD Biosciences), and the data were processed using FACS Diva 8.0.1 software. Isolated bone marrow (BM) cells from femur and tibia of mice and peripheral blood (PB) collected from heart were initially treated with ACK lysing buffer (Thermo Fisher Scientific) to remove red blood cells. Then, flow cytometry analysis of GFP positive cells from BM and PB were assessed by MACSQuant and FlowJO software. Viable cells were discriminated from dead cells by DAPI exclusion.

### Cryosections

Organs or embryos (E10.5) were fixed with 4% PFA-PBS, washed twice with PBS and treated with 30% Sucrose for 48 hours at 4 °C. They were then embedded in OCT (optimal cutting temperature) compound and sectioned (10 µM thickness). Slides were washed once with PBS, incubated with DAPI (4′, 6-diamidino-2-phenylindole) (1 µg/ml) for 10 min, washed again with PBS and mounted using ProLong Antifade solution (Molecular probes, Invitrogen).

## Electronic supplementary material

Supplementary Material
