# Supplementary material for: A single reporter mouse line for Vika, Flp, Dre, and Cre-recombination
Source: Sci Rep. 2018 Sep 27;8:14453. doi: 10.1038/s41598-018-32802-7 (PMC6160450; doi:10.1038/s41598-018-32802-7)
Supplement: Supplementary file 1 — Supplementary Material [file 41598_2018_32802_MOESM1_ESM.pdf]

Supplementary Material

A single reporter mouse line for Vika, Flp, Dre, and Cre-recombination

Madina Karimova[1*§], Oliver Baker[2*¶], Aylin Camgoz[1], Ronald Naumann[3], Frank Buchholz[1,3 #] & Konstantinos Anastassiadis[2#]

Supplementary material contains 9 supplementary figures and 2 suppl. Tables

Figure S1 is related to Figure 1
Figure S2.1 is related to Figure 2
Figure S2.2 is related to Figure 2
Figure S2.3 is related to Figure 2
Figure S3.1 is related to Figure 3
Figure S3.2 is related to Figure 3
Figure S3.3 is related to Figure 3
Figure S4.1 is related to Figure 4
Figure S4.2 is related to Figure 4

Table S1: Cloning Oligo’s
Table S2: Genotyping primers

Supplementary Figure S1

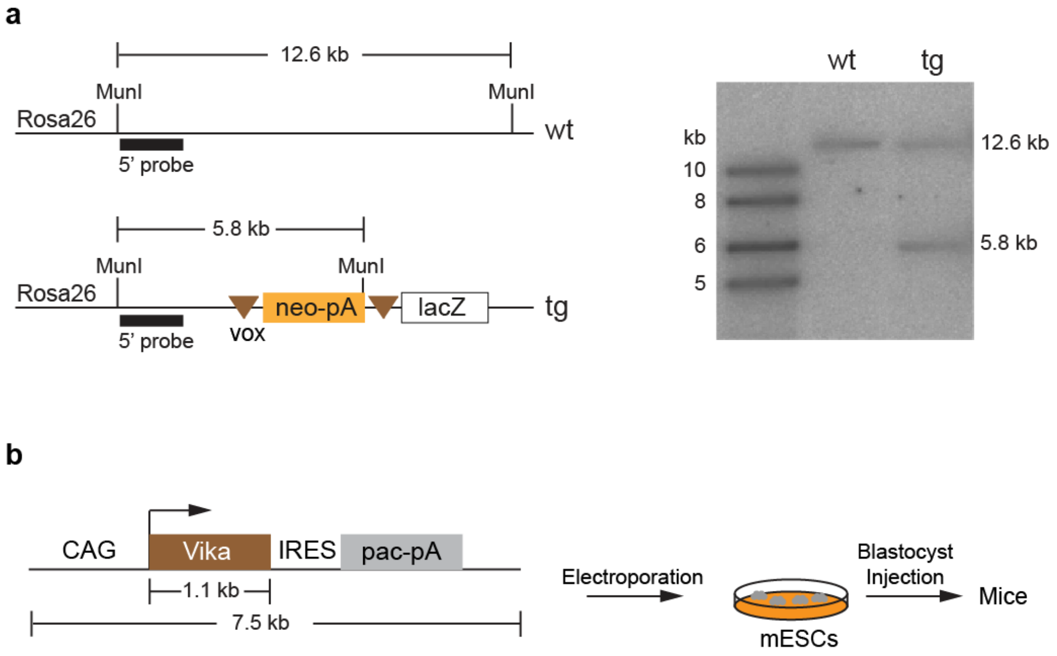

Supplementary Figure S1. (**a**) Schematic diagram of the Rosa26 locus before and after targeting of the Vika-reporter lacZ cassette. The right panel shows a Southern blot using mESC gDNA (clone #3) digested with MunI and hybridized with an external Rosa26 5' probe. (**b**) Generation of Vika deleter mice. The CAG-Vika-IRES-puromycin-pA vector was linearized with SpeI and electroporated into R1 mESCs. Puromycin resistant clones were checked by Southern blot for single integration events and by transient transfection of the reporter for functionality. Two clones were selected for injection into blastocysts and one of them gave germline transmission.

Supplementary Figure S2.1

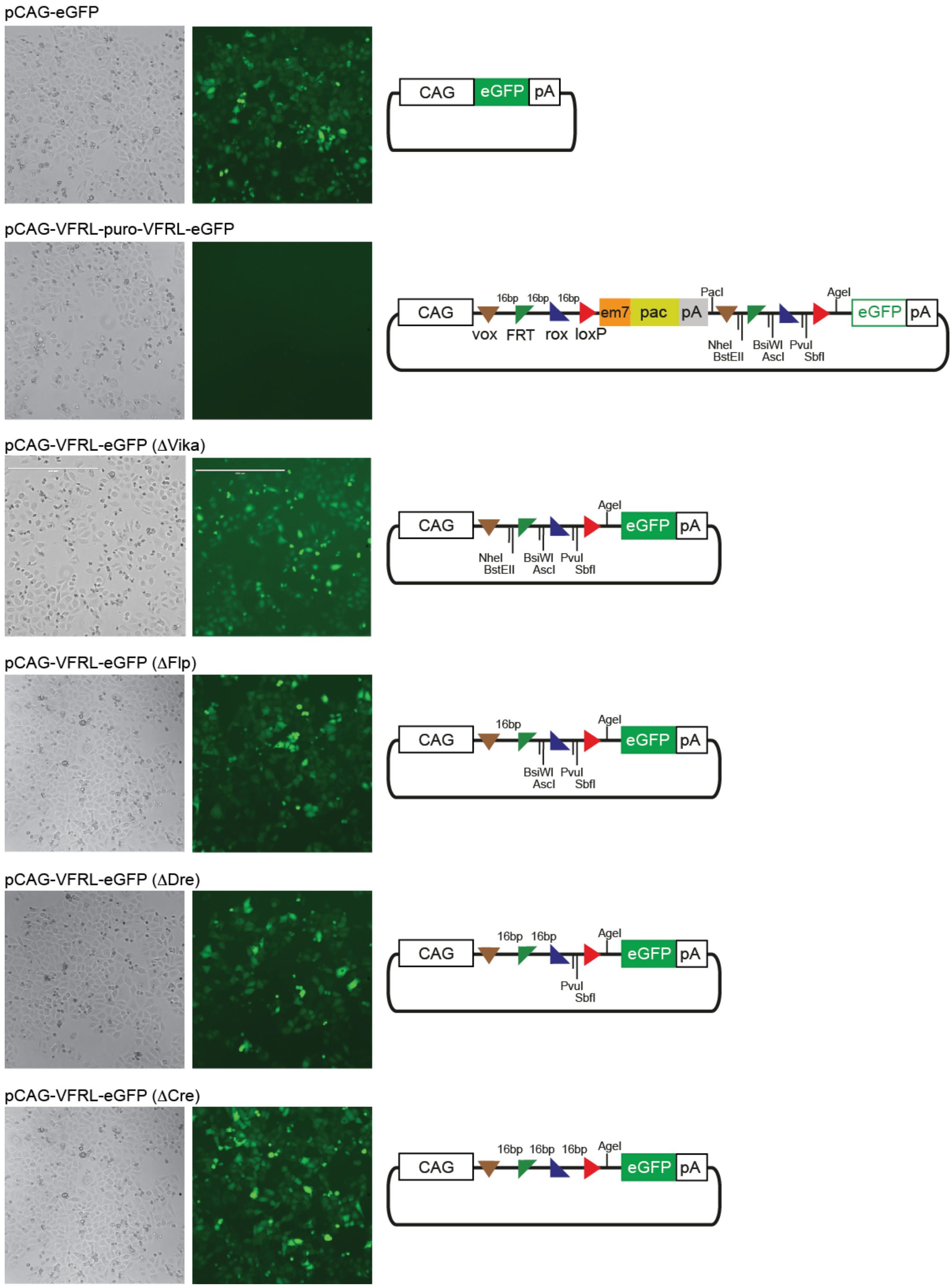

Supplementary Figure S2.1. Validation of the multi-site reporter construct by transient transfection of *E.coli* pre-recombined versions into HeLa cells. Scale bar = 400 µm.

Supplementary Figure S2.2

Sequencing of VFRL reporter recombined by **Vika** in *E.coli*

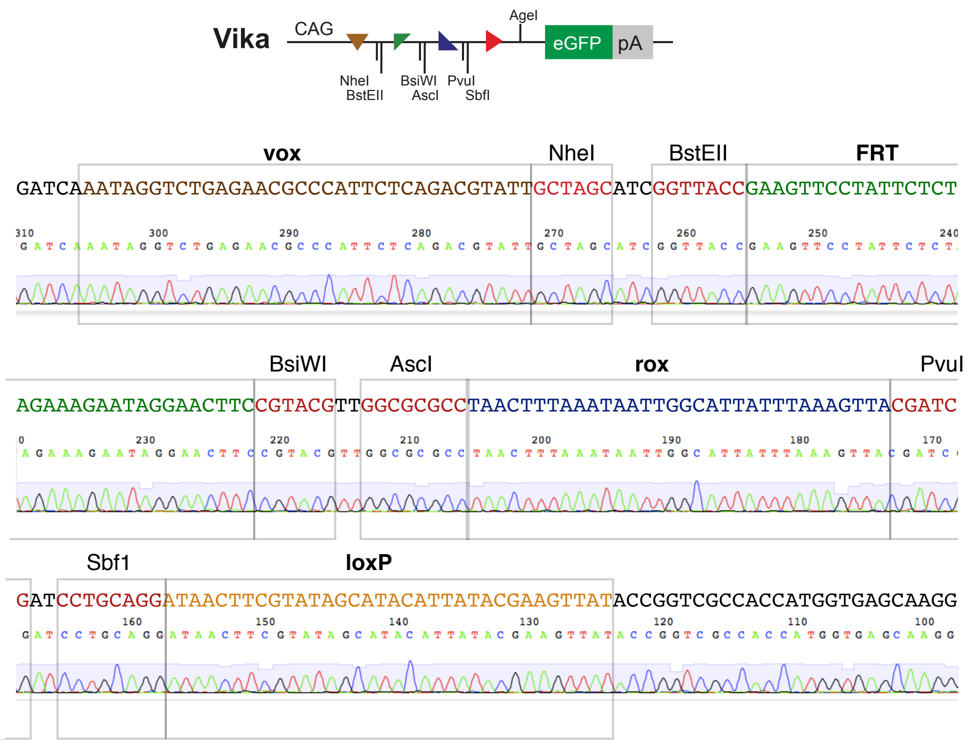

Sequencing of VFRL reporter recombined by **Flpo** in *E.coli*

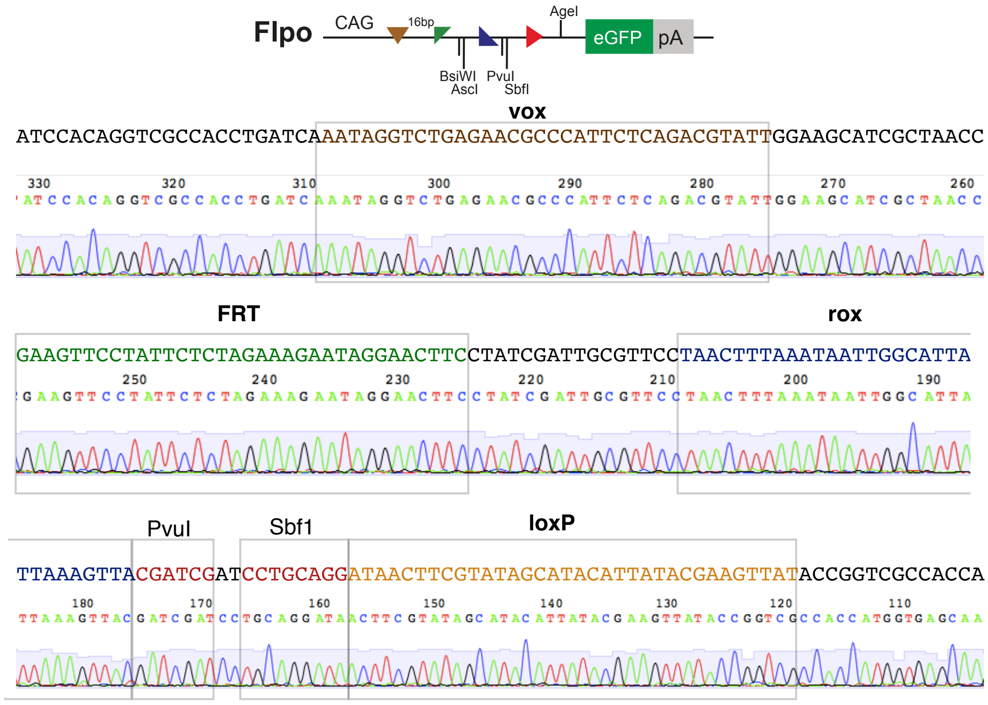

Supplementary Figure S2.2 (continue)

Sequencing of VFRL reporter recombined by **Dre** in *E.coli*

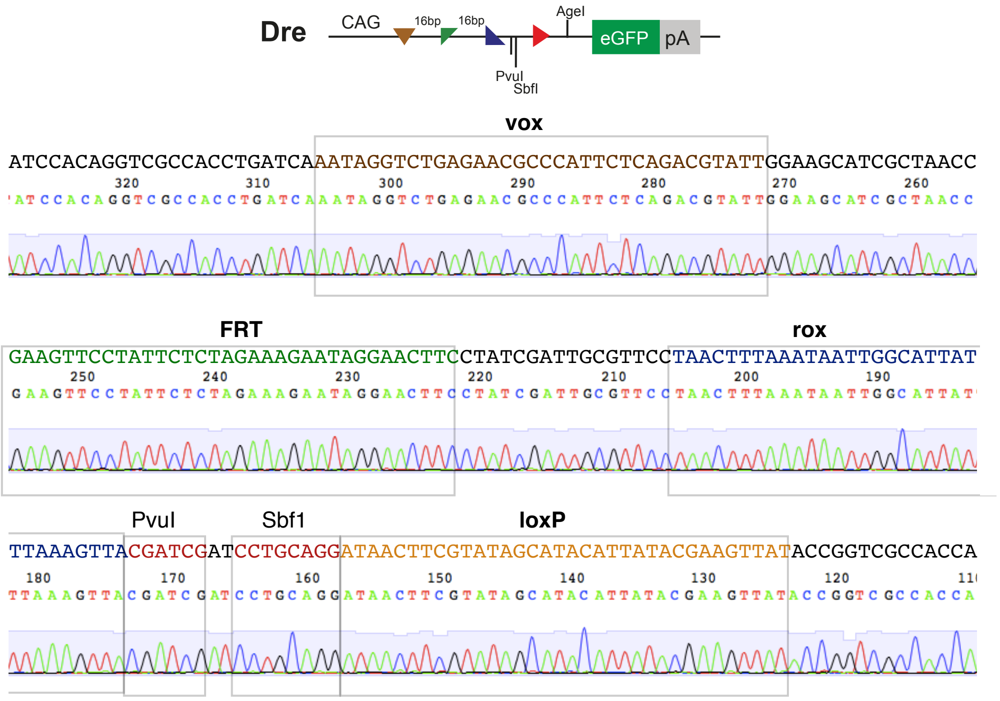

Sequencing of VFRL reporter recombined by **Cre** in *E.coli*

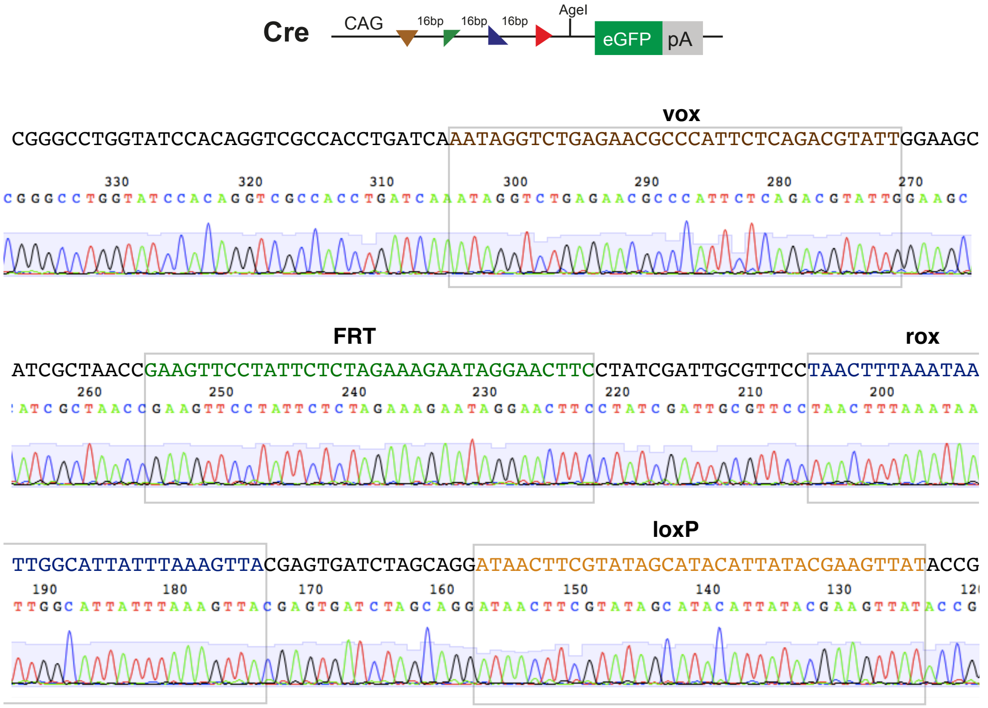

Supplementary Figure S2.2. Sequencing results of the recombined multi-site reporter after co-transfection with each one of the 4 recombinases. The recombined products were PCR amplified across the RTs and sequenced. A diagram of the recombined product is shown above each sequence. Each recombinase generates a specific genetic fingerprint, which was confirmed by sequencing. Recombination target sites and endonuclease restriction sites are marked by open rectangular.

Supplementary Figure S2.3

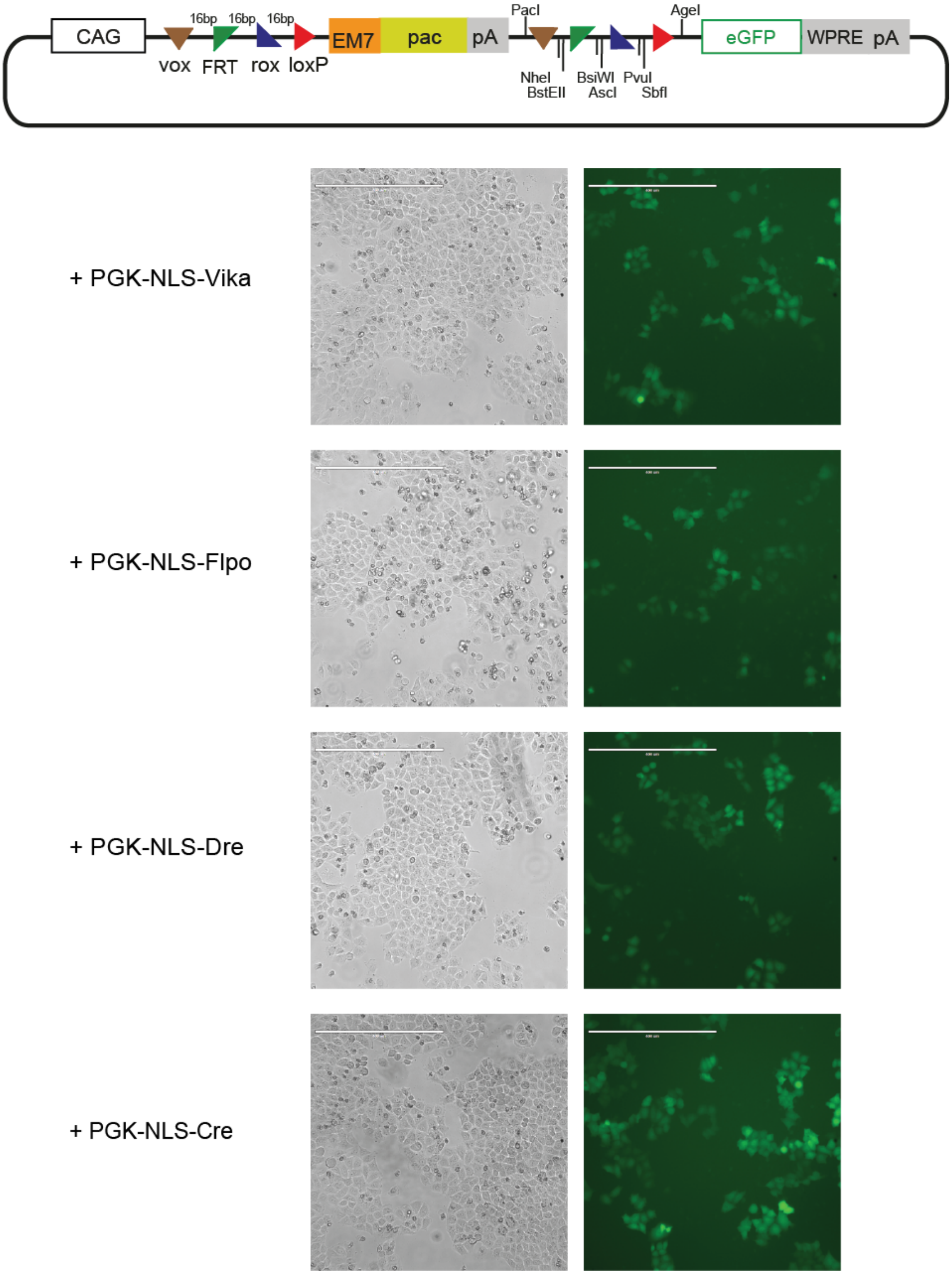

Supplementary Figure S2.3. Validation of the multi-site reporter before addition of the nuclear localization signal (NLS) to eGFP in HeLa cells. The multi-site reporter was co-transfected with Vika, Flpo, Dre and Cre recombinase expression vectors under the control of the PGK promoter in HeLa cells.

Supplementary Figure S3.1

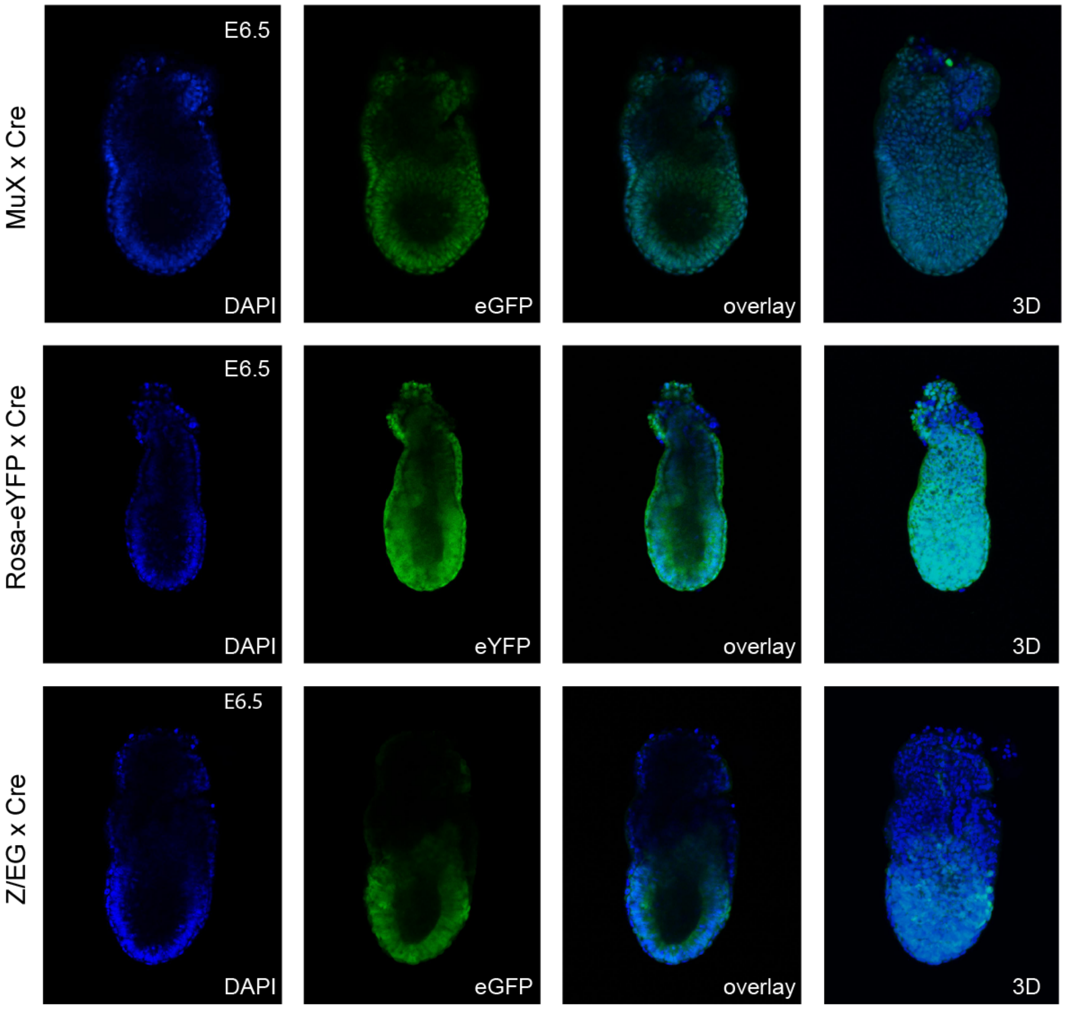

Supplementary Figure S3.1. Micrographs showing embryos at E6.5 isolated from intercrosses between PGK-Cre deleter male mice and Z/EG, Rosa26-eYFP and MuX reporter mice. Note the nuclear localization of eGFP in all cells of the MuX reporter embryo. The panels from left to right show DAPI staining of the nuclei, Fluorescent protein expression, overlay and 3D reconstruction of the embryo. Imaging was done with a Zeiss LSM 780 inverted confocal microscope. (eYFP was imaged using eGFP conditions - all images were taken at the same magnification).

Supplementary Figure S3.2

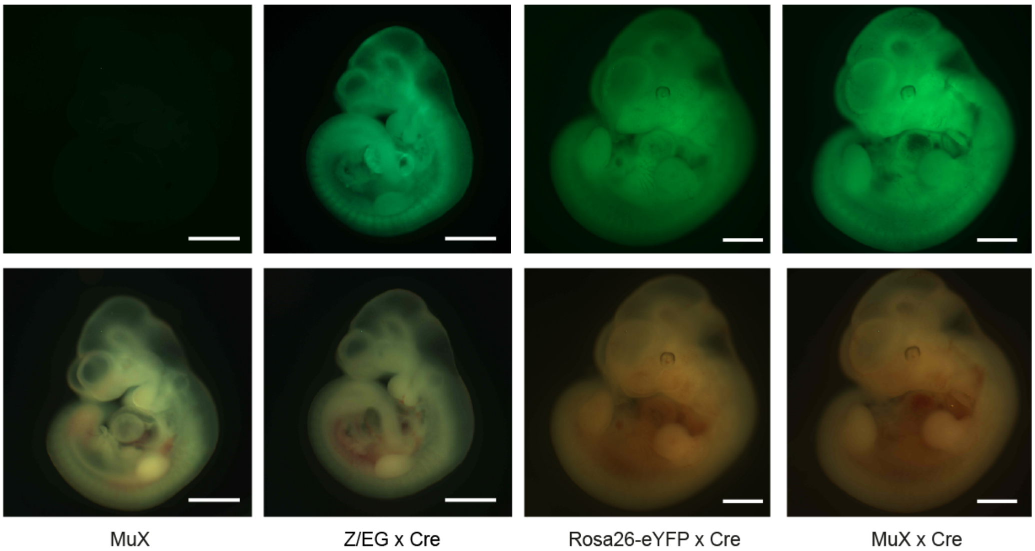

Supplementary Figure S3.2. Micrographs showing embryos at E10.5 isolated from intercrosses between PGK-Cre deleter male mice and Z/EG, Rosa26-eYFP and MuX reporter mice. The reporter protein (eYFP or eGFP) is ubiquitously expressed. Scale bar = 1 mm.

Supplementary Figure S3.3

a

Cre x Z/EG (2 litter, n = 12)

Cre +	Z/EG +	Cre + Z/EG +	Rec.
7	12	7	7

Flpo x RC::Fela (1 litter, n = 9)

Flpo +	Fela +	Flpo + Fela +	Rec.
3	5	2	2

Dre x Rosa-rox-LacZ (1 litter, n = 7)

Dre +	RrLZ +	Dre + RrLZ +	Rec.
3	4	3	3

Vika x Z/EG (4 litter, n = 13)

Vika +	Z/EG +	Vika + Z/EG +	Rec.
11	13	11	0

Vika x RC::Fela (2 litter, n = 12)

Vika +	Fela +	Vika + Fela +	Rec.
3	5	2	0

Vika x Rosa-rox-LacZ (1 litter, n = 9)

Vika +	RrLZ +	Vika + RrLZ +	Rec.
6	5	4	0

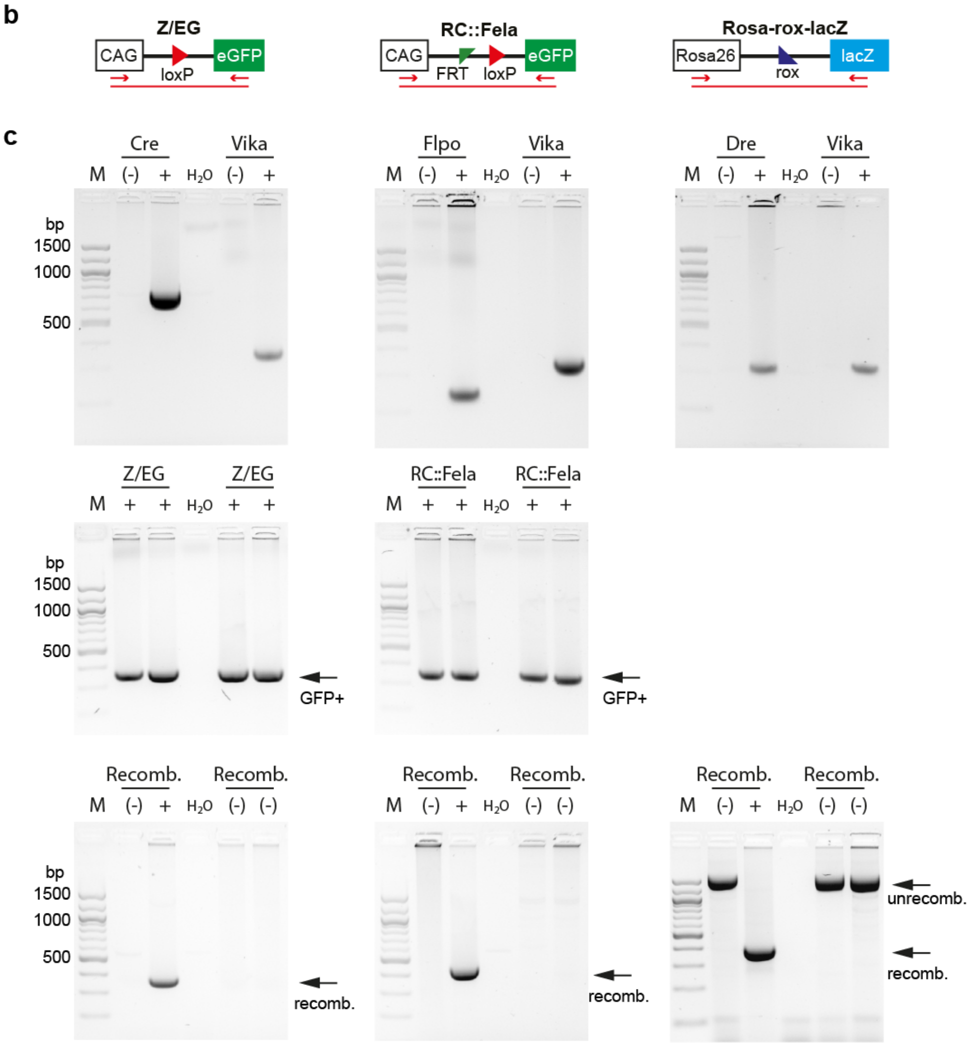

Supplementary Figure S3.3. Intercrosses between Vika deleter and Cre (Z/EG), Flp (RC::Fela) and Dre (Rosa-rox-lacZ) recombinases reporter mice. (**a**) Genotyping results from each intercross. The number of E10.5 isolated embryos for each recombinase, respective reporter and double positive are indicated. (**b**) Schematic diagram of the recombined loci Z/EG, RC::Fela and Rosa-rox-lacZ. Arrows show the PCR primers placed upstream and downstream of the target sites for detecting recombination (recomb.). (**c**) Representative PCR results for the presence of the respective recombinase (upper panels), presence of the reporter (middle panels) and recombination (lower panels). For Dre-recombination the PCR conditions are optimized for detecting unrecombined and recombined products with the same primers. There was no recombination between Vika x Z/EG, Vika x RC::Fela and Vika x Rosa-rox-lacZ embryos. M = molecular weight marker.

Supplementary Figure S4.1

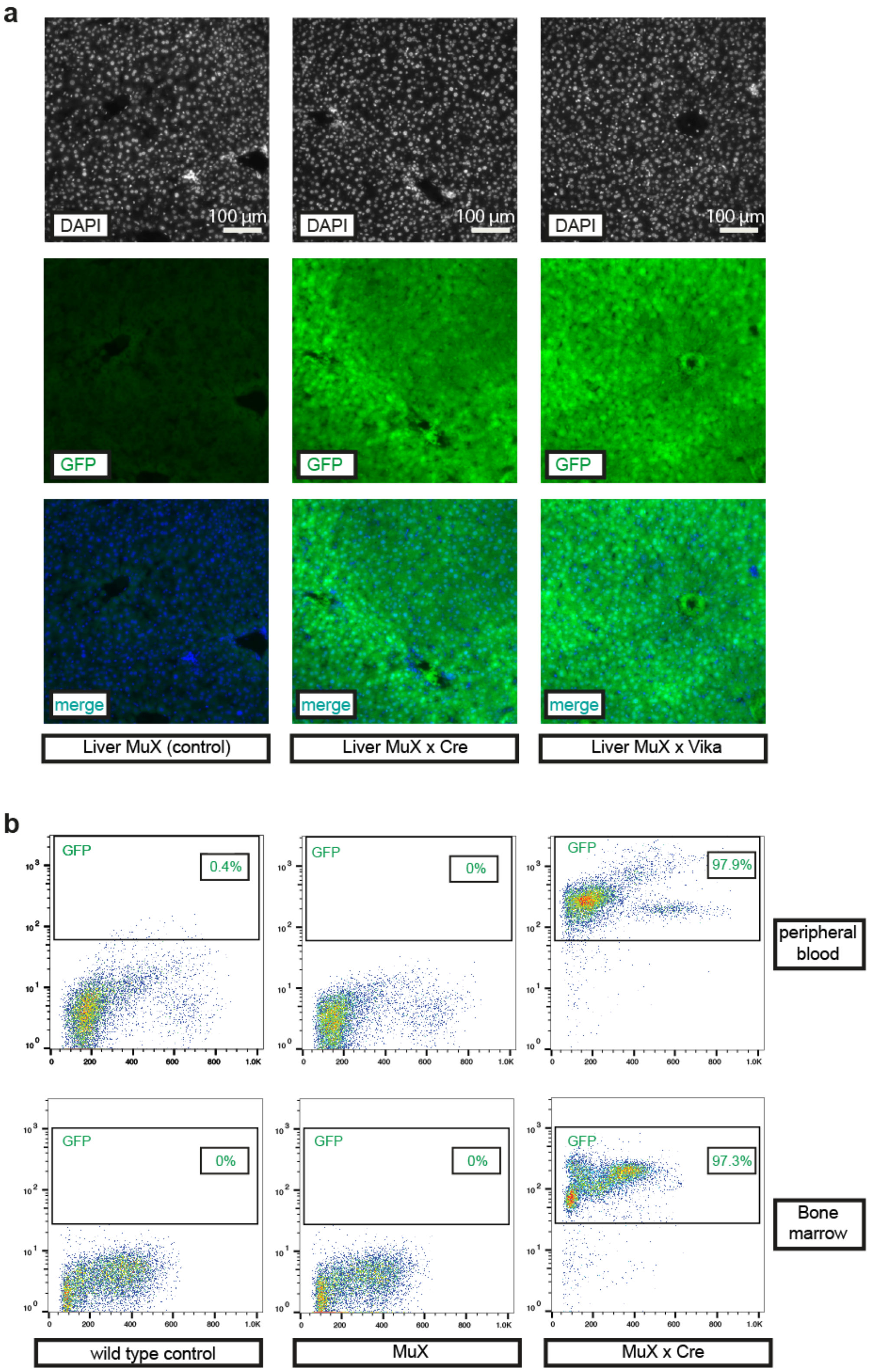

Supplementary Figure S4.1: (a) Liver cryosections (10 µm) from MuX control mice, MuX x Cre and MuX x Vika stained with DAPI and photographed with a NICON Eclipse Ti-S microscope. Scale bar = 100 µm. (**b**) GFP expression was analysed by flow cytometry in peripheral blood (upper panels) and bone marrow (lower panels) of MuX x Cre mice and controls.

Supplementary Figure S4.2

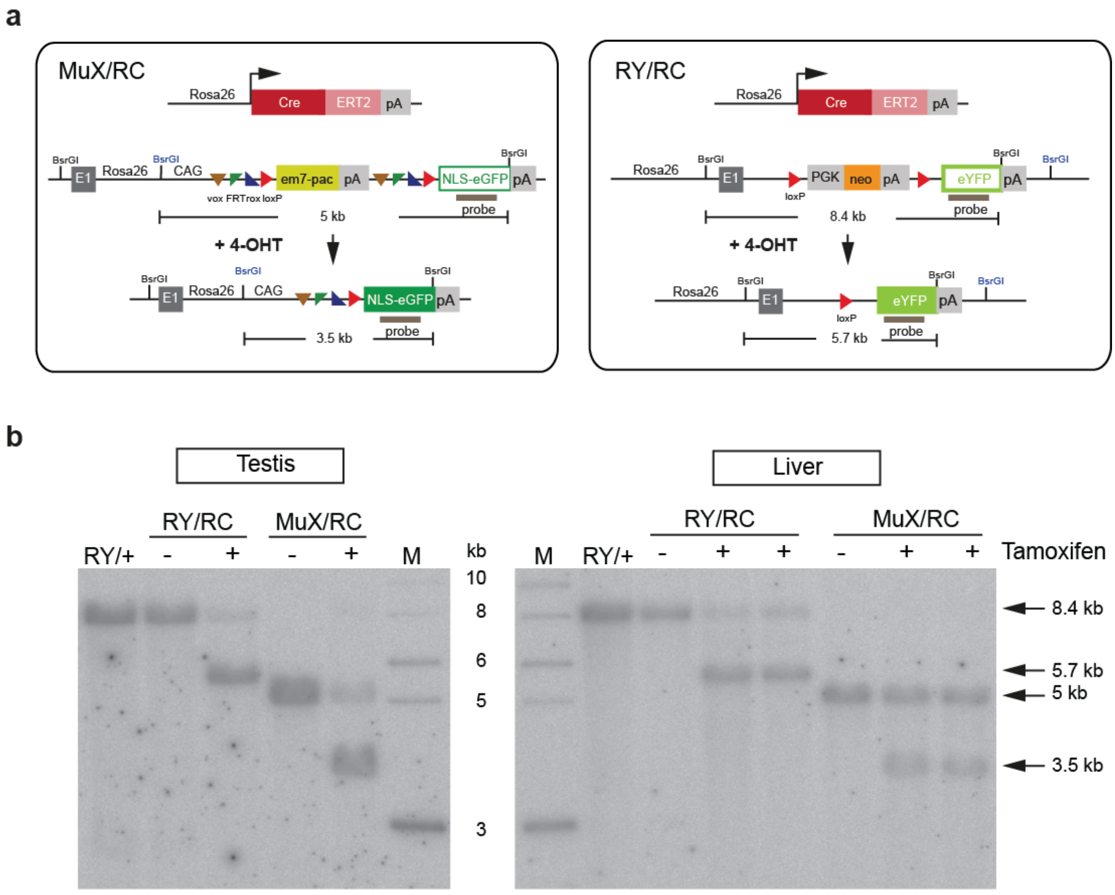

Supplementary Figure S4.2: Tamoxifen induced Cre-recombination in adult mice. Rosa26-Cre-ERT2 mice were crossed to MuX and Rosa26-eYFP reporter mice. **(a)** Schematic diagram of the MuX reporter (left) and Rosa26-eYFP reporter (right) before and after recombination. Please note that the insertion of the MuX-reporter cassette into the intron of the Rosa26 locus is 2784 bp downstream of Exon1 whereas the insertion of the eYFP-reporter cassette is 1031 bp downstream of Exon1. Those differences in the two reporter loci are indicated in the diagrams by the BsrGI site marked in blue. (**b**) Southern hybridization analysis using a GFP probe and DNA from testis (left) and liver (right) from single reporters (Rosa26-eYFP = RY/+) and double knock-in (KI) mice (RY and Rosa26-Cre-ERT2 = RY/RC or MuX and RC = MuX/RC) with (+) and without (-) tamoxifen. Two double KI mice (one male and one female) of each genotype were induced. M = molecular weight marker.

Supplementary Table S1: Cloning oligo's

Oligo name	Sequence (5' to 3')
Vika-Fwd	GATCGAATTCCACCATGAAGAAAAAGCGGAAAGTGACC
Vika-Rev	GATCGAATTCCTAGACTCTAGACCGCTGTCTCCGC
Rosa-vox-neo-fwd	CTGGGCAACGTGCTGGTTATTGTGCTGTCTCATCATTTTGGCAAAGAATTCAATAGGTCTGAGAACGCCCATTCTCAGACGTATTAGCACGTGTTGACAATTAATC
Rosa-vox-neo-rev	GACTGTCGATTGGACTCAGGAGTGTAGTGATGGAGCCGGAGACGGTTACCAAATACGTCTGAGAATGGGCGTTCTCAGACCTATTGATCCAGACATGATAAGATAC
NLS-eGFP-up	TATACCGGTCGCCACCATGGCTCCTAAGAAGAAAAGGAAGGTGGTGAGCAAGGGCGAGGAG
eGFP-BsrGI-low	ACTTGTACAGCTCGTCCATGCCGAGAG
WPRE-up	ACTTGTACAAGTAAGCGATCGCTTTACGCTATGTGGATACGCTGC
WPRE-low	AGCGTACGTTACTACGCGTAAAGGGAGATCCGACTCGTCTG

Supplementary Table S2: Genotyping primers

Primer name	Sequence (5' to 3')	Annealing / Product size (bp)	Application
eGFP-F<br>eGFP-R	CTTCTTCAAGGACGACGGCAACTA<br>ATCGCGCTTCTCGTTGGGGTCTTTGC	58°C / 355	eGFP genotyping
19 se<br>20 as	GCCTGCATTACCGGTCGATGCAACGA<br>GTGGCAGATGGCGCGGCAACACCATT	67°C / 700	Cre genotyping
Flpo3<br>Flpo4	GATCACCGAGAAGATCCTGAAC<br>CTCTGGCGCTGAAAAAGTAGAT	58°C / 242	Flpo genotyping
Dre3<br>Dre4	TGCTGTTCCCTCCTATCCAC<br>CGGAGTCCATCAGCCTAGAG	58°C / 314	Dre genotyping
Vika-F1<br>Vika-R1	AGGACGTGGAAAGATACCTGAT<br>ATGTCGCCCACTTTCATCTGTT	60°C / 346	Vika genotyping
CAG-seq1<br>eGFP-seq5	AGCCTCTGCTAACCATGTTCATG<br>ATGAACTTCAGGGTCAGCTTG	58°C / 557 (MuX)	MuX, Z/EG, RC::Fela recombination
CoCT-5out<br>eGFP-seq5	CAGACCCTTGTCTTACACCAT<br>ATGAACTTCAGGGTCAGCTTG	58°C / 495	MuX incomplete recombination
Rosarox1<br>Rosaroxneo1	TGGAAATGTTACCAAGGAACT<br>TGACAGGAGATCCTGCCCCGGCACT	58°C / 716	Rosa-rox genotyping
Rosarox1<br>RosaroxLZ1	TGGAAATGTTACCAAGGAACT<br>AACGACGGCCAGTGCCAAGCTACT	58°C / 405 unrecomb. = 1850 bp	Dre (Rosa-rox) recombination
